# Acceptability of home-based transcranial direct current stimulation (tDCS) in bipolar depression: thematic analysis of individual views

**DOI:** 10.1186/s12888-025-06948-4

**Published:** 2025-05-26

**Authors:** Hakimeh Rezaei, Rachel D. Woodham, Ali-Reza Ghazi-Noori, Philipp Ritter, Michael Bauer, Allan H. Young, Elvira Bramon, Cynthia H.Y. Fu

**Affiliations:** 1 Department of Psychiatry and Psychotherapy, Faculty of Medicine, Technische Universität Dresden, Dresden, Germany; 2 Centre for Affective Disorders, Department of Psychological Medicine, Institute of Psychiatry, Psychology and Neuroscience, King’s College London, London, UK; 3 Department of Psychology, University of East London, London, UK; 4 Department of Psychiatry, University College London, London, UK; 5 National Institute for Health Research Biomedical Research Centre at South London and Maudsley NHS Foundation Trust, King’s College London, London, UK; 6 South London and Maudsley NHS Foundation Trust, Bethlem Royal Hospital, Beckenham, UK

**Keywords:** Thematic analysis, Acceptability, Qualitative analysis, Transcranial direct current stimulation, Home-based treatment, bipolar depression

## Abstract

**Background:**

Acceptability is a multifaceted concept that reflects how a treatment is viewed, which impacts patient engagement, adherence, and provider implementation. Transcranial direct current stimulation (tDCS) is emerging as a novel non-invasive treatment for bipolar depression. We developed a home-based protocol for tDCS, which has demonstrated efficacy in unipolar and bipolar depression. We sought to explore the acceptability of home-based tDCS in bipolar depression.

**Methods:**

Participants were 35 adults (26 women) with bipolar disorder (mean age 47.37 years, SD ± 13.78) in a current depressive episode of at least moderate severity. tDCS was provided in a bifrontal montage, 2 mA for 30 min each session, over 6 weeks with real-time supervision. Acceptability was assessed in a questionnaire and individual interviews, conducted at two timepoints: baseline and post treatment. Individual interviews were analysed by thematic analysis.

**Results:**

Six main themes were found: helpfulness, side effects, burden, gratitude, ethicality and comparison to medications. The themes of gratitude and comparison with medications were novel in this group compared to unipolar depression.

**Conclusion:**

Themes reflected high acceptability of tDCS treatment in bipolar depression and indicated strong interest in novel treatments in this population. Qualitative analysis can provide novel insights into individual experiences, understand barriers to treatment, and offer guidance for improving clinical treatments.

**Trial registration:**

ClinicalTrials.gov: NCT05436613 registered on 23 June 2022 https//www.clinicaltrials.gov/study/NCT05436613.

**Supplementary Information:**

The online version contains supplementary material available at 10.1186/s12888-025-06948-4.

## Background

Acceptability refers to the extent to which individuals perceive a treatment as fitting and appropriate, whether they are delivering or receiving it. This perception is shaped by both their expectations and experiences, including emotional and cognitive reactions [1–3]. Treatments that are clear, easy to use, and align with patients’ values and perceived efficacy are more likely to be considered acceptable. When treatments are well-received and acceptable, patients tend to participate more actively, follow prescribed recommendations, and achieve better health outcomes [4].

Sekhon et al. [5] developed a Theoretical Framework of Acceptability (TFA) to address the need for a comprehensive understanding of acceptability in healthcare interventions. Their work aimed to provide a clear definition and structure for assessing how acceptable an intervention is to those delivering or receiving it. The framework identifies seven key constructs that influence acceptability: (1) affective attitude, referring to the individuals’ feeling toward the intervention; (2) burden, describing the effort and resources required to participate in the intervention; (3) perceived effectiveness, the degree to which the intervention is believed to achieve its desired outcomes; (4) ethicality, the compatibility of the intervention with personal values and moral standards; (5) intervention coherence, the extent to which individuals understand how the intervention works; (6) opportunity costs, the benefits, profits, or values that must be given up to engage in the intervention; (7) self-efficacy, the confidence individuals have in their ability to successfully perform the behaviors required by the intervention.

Bipolar disorder is a mood disorder characterized by recurrent episodes of (hypo-) mania and depression. Episodes of depression are often more frequent and longer lasting than the manic episode [6]. Bipolar depression is associated with disability and functional impairment across various domains, including work or school responsibilities, household duties, and maintaining relationships [7, 8]. Furthermore, depressive episodes are associated with an increased risk of suicidal behavior, emphasizing the importance of effective treatments [9]. The treatment for bipolar disorder typically includes mood stabilizing medication, such as lithium and valproate, antipsychotic medications, such as quetiapine, and psychosocial approaches, including psychoeducation and family-focused therapy. However, challenges in bipolar depression include limited treatment options, adherence issues due to side effects (e.g., antipsychotic-induced weight gain or sedation), and the risk of manic switching with antidepressant use [10].

Transcranial direct current stimulation (tDCS) is a novel non-invasive technique used to modulate brain activity by delivering a weak current (1–2 mA) to the scalp via electrodes (anode and cathode) [11]. Meta-analyses of randomized sham-controlled trials have shown that tDCS significantly reduces depressive symptoms in bipolar disorder (SMD = -1.18, 95% CI: -1.66 to -0.69) and demonstrates a high response rate compared to sham [12] with longer protocols associated with greater clinical improvement [13]. Significant improvements in depressive symptoms have been observed, in which active tDCS was superior to sham at the treatment endpoint [14]. Protocols using 30-minute sessions at 2 mA are considered safe, tolerable, and effective for modulating cortical excitability and plasticity, and are commonly used across trials [12]. Affective switching into mania or hypomania is a critical safety concern in the treatment of bipolar depression, particularly with antidepressant therapies [12]. Although tDCS is generally considered to carrying a low risk of inducing a manic switch, monitoring for mood destabilization remains essential [15]. Notably, no treatment-emergent affective switching had been observed in recent trials and meta-analyses evaluating tDCS for bipolar depression [12, 15, 16].

tDCS trials had generally provided the treatment in a clinic or research centre. As the sessions are several times a week, this requires frequent visits to a clinical setting, potentially creating barriers to access. Due to its portability and safety, tDCS could be administered at home [17]. We developed a home-based tDCS treatment protocol in which participants use the tDCS device by themselves with real-time remote supervision by researchers using video conference [18]. Our randomised sham-controlled trial of home-based tDCS in unipolar depression observed a significant effect of active stimulation compared to sham in improvements in depressive symptoms, safety, and high acceptability at the 10-week end of treatment [19], and our open-label study in bipolar depression showed high safety and clinical outcomes [18].

We investigated the acceptability of home-based tDCS with remote supervision for bipolar depression. We developed acceptability questionnaires and semi-structured interviews based on Sekhon et al.’s [5] Theoretical Framework of Acceptability. Qualitative research provides insights into subjective experiences and contextual elements that are often beyond the reach of quantitative approaches. In healthcare settings, qualitative methods are particularly effective for assessing patient experiences, identifying obstacles to treatment, and enhancing the delivery of care [20]. In unipolar depression, we found four themes from individual interviews: effectiveness, side effects, time commitment and support, and feeling held and contained. These themes demonstrated the high acceptability of tDCS treatment among individuals with unipolar depression [21]. Here, we performed a qualitative analysis of individual experiences in participants with bipolar depression through interviews conducted at the end of treatment. We sought to investigate how home-based tDCS is experienced by individuals with bipolar depression and to assess potential novel factors that may influence acceptability in this population.

## Method/design

### Study design

The study was an open-label, single-arm acceptability and feasibility trial of home-based tDCS for bipolar depression (ClinicalTrials.gov: NCT05436613 registered on 23 June 2022 https//www.clinicaltrials.gov/study/NCT05436613). The study was conducted in accordance with the Code of Ethics of the World Medical Association (Declaration of Helsinki) and approved by the London Fulham Research Ethics Committee (21/LO/0910). Participants were recruited via online advertisements, referrals from general practitioners, psychiatrists and community mental health teams. After study details were explained and any questions were answered, informed written consent was obtained electronically or in written form for each participant by a research team member.

Assessment visits were conducted remotely in real-time by Microsoft Teams videoconference. Participants were also able to attend visits in person, but no participant chose to attend in person. Following a comprehensive clinical assessment, the tDCS device (Supplementary Materials Fig. 1) was sent to the enrolled participant by post. A research team member would show each participant how to use the device in real-time by Microsoft Teams video conference, including how to correctly position and adjust it. In addition, the device was connected to a mobile app that provided step-by-step instructions, including a camera-based mirror function with alignment guides to assist with electrode placement. The app confirmed correct positioning before each session began, helping to ensure consistent and accurate electrode placement across participants.

### tDCS protocol

The protocol consisted of 30-minutes active tDCS sessions in a bifrontal montage: the anode was placed at left DLPFC (F3 position according to the international 10/20 EEG system), and the cathode was placed at right DLPFC (F4 position) (Supplementary Materials Fig. 1). The stimulation was 2 mA for 30 min, with a gradual ramp up over 120 s at the start and ramp down over 15 s at the end of each session. There were 5 sessions a week for 3 weeks, then 2 sessions a week for another 3 weeks, totaling 21 sessions. A minimum of 15 sessions was required for study completion. A member of the research team was present during each session, providing a discreet presence with their camera on, while participants had both their camera and microphone enabled to facilitate communication if needed. Interaction between the participant and the team occurred only when the participant required assistance. During sessions, participants were allowed to read, use handheld devices, tablets, laptops, or desktop computers, or sit quietly.

Adults with bipolar depression experiencing a minimum of moderate severity of depressive symptoms as assessed in structured clinical assessments and clinician-rated depressive severity scales were eligible. Full inclusion and exclusion criteria are provided in Supplementary Materials.

### Acceptability questionnaire

The acceptability questionnaire was developed based on Sekhon et al.'s [5] Theoretical Framework of Acceptability and consisted of five questions centred on acceptability sub-facets:

1. Overall acceptability: ‘How acceptable did you find the tDCS sessions?’
2. Subjective efficacy: ‘How helpful were the tDCS sessions for improving your depressive symptoms?’;
3. Adverse effects: ‘How were you bothered by any negative side effects from the tDCS sessions?’;
4. Ethical perspectives: ‘How ethical do you think the tDCS sessions are?’;
5. Overall burden: ‘How much effort did you need to put in for the tDCS sessions?’.

Responses were assessed on a 7-point Likert style scale along with open-ended responses. Acceptability data were acquired at baseline and week 6 (the above questions in retrospective wording for the end of treatment assessment). An additional question and four open-ended questions were asked at the end of treatment assessment:

6. Recommendation: ‘Would you recommend the tDCS sessions to others?’;
7. Positive aspects: ‘Please explain, in your view, what were the most successful parts of the study?’;
8. Negative aspects: ‘Please explain, in your view, what were the least successful parts of the study?’;
9. Possible improvements: ‘Are there ways in which the study can be improved?’;
10. Further comments: ‘Do you have any other comments?’.

Participants completed the questionnaire during a semi-structured interview, based on Kallio et al. [20], which was recorded on video using Microsoft Teams. The interviewers were the same researchers who were present during each trial session. Participants were encouraged to speak openly about their experiences. Recorded interviews were then transcribed verbatim. Written or Likert-only responses were excluded from the thematic analysis.

### Quantitative thematic analysis

Thematic analysis was employed to systematically identify, analyze, and report patterns within the data, capturing and describing key aspects of participants’ experiences and perspectives [22]. Thematic analysis typically involves six steps. The full methodological steps and coding process are detailed in Supplementary Materials.

## Results

A total of 44 bipolar depression participants (31 women) were enrolled in the study. Three participants did not attend the final session, two were unable to connect due to technical issues with the video link, and four declined to be recorded for the interview. As a result, 35 participants (26 women) (mean age 47.37 ± 13.78 years) completed post-treatment acceptability interviews. At baseline, the mean MADRS score was 24.77 ± 2.72, reflecting severe depressive symptoms, and at the end of treatment, the mean MADRS score was 7.33 ± 4.43 (Supplementary Materials, Table 1).

The quantitative analysis of the results, including clinical ratings and Likert acceptability questionnaire responses, has been reported separately [18]. Overall, 93.2% of participants (*n* = 41) completed the full 6-week course of treatment, and 72.7% of participants (*n* = 32) completed the 5-month follow-up. The treatment was associated with significant clinical improvements from baseline (mean MADRS 24.78 ± 3.00) to the 6-week end of treatment (mean MADRS 8.13 ± 5.48, F_(2,62)_ = 80.30, *p* < 0.001), which were maintained at the 5-month follow-up (mean MADRS 10.81 ± 7.46). There was a notable shift in the endorsement of acceptability, with participants rating it as “quite acceptable” at baseline and transitioning to “very acceptable” at the end of treatment and at follow-up. Ratings for perceived effectiveness were endorsed as being “quite helpful” at baseline and end of treatment and “very helpful” at follow-up, with no significant change over time [18].

Adverse effects were mild and transient in over 90% of cases, with the most common being tingling, skin redness, itching, and burning sensations, which are typical side effects of tDCS [23]. The impact of side effects significantly decreased over time, improving from being “a bit unaffected/quite unaffected” at baseline to being “very much unaffected” post-treatment and “very much unaffected/quite unaffected” at follow-up. There were no serious adverse effects or instances of treatment-emergent affective switching [18].

The thematic analysis of individual interviews revealed six overarching themes, each capturing distinct yet interconnected aspects of participants’ experiences. These themes are supported by direct quotes from participants to illustrate their experiences [24] (Supplementary Materials, Table 2).

### Theme 1: helpfulness

The first theme reflects participants’ perceptions of the treatment’s impact, highlighting its effectiveness or limited effectiveness, acceptability, and unique aspects, as well as varying experiences of improvement and confidence in the treatment. There were four subthemes: (1a) effectiveness and acceptability, (1b) unexpected improvement, (1c) gradual improvement, and (1d) novelty and un/certainty.

### Subtheme 1a: effectiveness and acceptability

Participants frequently reported that the treatment was effective in improving symptoms:

All of it really made me… it made me feel a person again. I wasn’t just floating under this black cloud. I’ve now like, blossomed again and… I’m talking to my family again and… it’s just everything has changed [Participant D, Female].

I thought that I was incredibly impressed with how good it was. Um I was stuck in a a a very low place when I started to do this test. I can’t take antidepressants because they give me mania and I felt like I didn’t have any hope that there was nothing that I could try to improve these depressive symptoms. But within 10 days or so of doing this, I felt a a big lift in my base level. I didn’t have anymore suicidal thoughts. I stopped catastrophizing. it just really helped lift me of the… bottom of the of the low mood that I was in [Participant Z, Female].

While some experienced significant improvement, others noted only slight or no changes:

That not, not really (laugh) I haven’t really seen any anything changes. Not very helpful. Yeah [Participant H, Female].

### Subtheme 1b: unexpected improvement

Some participants mentioned that they initially had different expectations for the treatment:

I wasn’t like expecting this. I was, I was like…I want to get a better, but I was thinking like it’s just the machine and it’s like really small. You know, it looks like simple and… But the result is like um really shock. Uh, because I had lots of many…depression in my life and it took like one and half year. But this time several months. I’m like is it helps me a lot. I’m so feeling lucky (short laugh) to find it (short laugh) [Participant F, Female].

### Subtheme 1c: gradual improvement

Few participants reported feeling a gradual improvement:

[…] and yeah, I didn’t expect as much improvement as I perhaps…ended up having. It was so gradual and that I didn’t really realize it was happening. And then kind of when I felt better and I looked back on it. It’s like, wow, can I really attribute it to just these sessions? like 5 days a week? It was kind of I didn’t expect them to be that successful [Participant R, Female].

### Subtheme 1d: novelty and un/certainty

Some participants highlighted the unique and innovative nature of the treatment and reported a certain degree of perceived effectiveness, while others were uncertain whether the improvement was actually due to the treatment:

I think they were helpful. I don’t know if it’s just a due to the sessions or some other… Ohh, changes in life but I’m feeling definitely feeling improvement [Participant E, Female].

I think it’s a little too early to tell. Umm, I do feel more cheerful as I said, but it could be because of other things. My mood is always fluctuated, so I think if there is a change, it’s subtle. Its effect effectiveness is subtle [Participant AH, Female].

[… ] you know this, this this is a new… something new that I’ve been able to try, but at the most positive thing. Is having something that isn’t another medication, that isn’t another therapy session or a mindfulness group or something something completely new that that I feel so far has had a positive result, and I hope it will continue to offer that [Participant L, Male].

### Theme 2: side effects

The second main theme addresses side effects related to the tDCS device, with two subthemes: (2a) side effects of the device and tolerability, and (2b) temporary side effect or adaptation.

### Subtheme 2a: side effects of the device and tolerability

Participants reported experiencing minor side effects caused by the device. However, none of the participants had described these effects as being significant, and some participants described that they did not experience any side effects. Nevertheless, the treatment was considered tolerable:

No, not at all. I thought I might get headaches, but I didn’t. No, absolutely fine. Nothing. I wouldn’t say any real side effects at all [Participant G, Female].

No, I wouldn’t say I’m both I was bothered. It was just there and with um… the app that… you know shows you what a thing, what what it feels like and with the brief…Um from you about um what to expect. Um…Yeah, it wasn’t. It wasn’t very much. I mean, it was like prickly was tingling, but. Yeah, that was it. So… [Participant Q, Female].

### Subtheme 2b: temporary side effect or adaption

Participants experienced adjusting to the device over time, with initial challenges often diminishing as they became accustomed to the treatment. Any side effects, if present, were temporary and diminished after a short period:

No, I have not. I mean initially there was some some…umm itching slight burning sensation umm some some redness to the scalp. But really as the study progressed those symptoms dissipated and I was barely aware of them at all and said it did not have any side effects after the sessions [Participant H, Female].

Umm so the main side effects that I noticed were the itching and tingling and during the sessions and but it wasn’t painful and or and didn’t bother me after the session um… I got used to it very quickly um as I did more and more sessions. um, I think I did experience skin redness after every session and like slightly uneven skin, and that took quite a long time, maybe two or three hours to disappear each time [Participant P, Female].

Just during like my skin was a little bit sore after but it would go away within the next like 20 min or something [Participant D, Female].

### Theme 3: burden

This theme describes the challenges participants encountered during the treatment, focusing on both the usability of the technology and the effort required to integrate the sessions into their daily lives. There were three subthemes: (3a) technical usability, (3b) time commitment, and (3c) treatment setting.

### Subtheme 3a: technical usability

This subtheme reflects participants’ experiences with the technical aspects of the device and the app. Many participants found the technology used in this study to be user-friendly and straightforward, though some mentioned initial or slight challenges with setup or operation:

[…] I can’t remember how often it happened. Sometimes you open up the app and it wouldn’t let you start it until you answered the…questionnaire and I was like…Ohh but I’m ready. And that was like. If they could give more warning or if they told you, hey, tomorrow’s session, we’re gonna ask you a very quick…the I can’t remember, was it 9 questions? Um…that, yeah, kind of annoyed me. I was like, I don’t wanna do this now and I’m ready (short laugh) So. that’s not your fault. That’s the app maker, I guess. […] [Participant T, Male].

### Subtheme 3b: time commitment

Many participants found the duration of the treatment to be short and manageable, as it easily fit into their lives, with some benefiting from the daily routine. However, a few experienced challenges with the commitment, such as fitting it into a busy schedule or finding it time-consuming:

[…] and the fact that it’s only 30 min a day…umm to begin with, and then less often means that it you can fit in around…umm your daily activities [Participant P, Female].

I think they’re very acceptable. They they fit it in my…in my normal life um I setting whilst doing the session itself I could carry on with my normal work and have activities and it was good that I could arrange…time that suited both the researcher and myself so, so, uh, there was no imposition on on either of our times and other than their longer session when we had some assessments where we’re usually wrapped up within half an hour, which was just allowed me to get on with the rest of my day [Participant V, female].

Um…getting back from work in time for them was sometimes challenging, but yeah, they certainly the the day-to-day half hour sessions were easy to fit in. obviously a little bit more time had to be put aside for the first, a bit at the beginning and a bit for Today. Um and fitting that in this been a little bit more challenging. Yep. OK in whole [Participant AA, Female].

A very well, very well. In fact it it gave me some sort of because I’m retired. It gave me some structure and I think that was one of the. The positives. Umm…whether it was the TCBS or or just having regular. Regular times when somebody was was was was concerned about it. You know, something was being done about about the [Participant A, Female].

### Subtheme 3c: treatment setting

This subtheme reflects participants’ experiences with the treatment setting. Many participants highlighted the convenience of having sessions at home; however, some preferred to follow their own schedule or attend in-person sessions:

Well, it was good in that it it taxed me a bit because I’m not very good on technology. But obviously it was. It was a great advantage of of not having to get myself somewhere else. You know, it’s very convenient to do everything from your own home [Participant A, Female].

I found them. Yeah, very easy. And being able to at home is obviously excellent [Participant G, Female].

I mean…Maybe face to face consultations are better. Maybe being able to go to a local. Umm…You know to, to, to, to, to go and be able to get the the treatment from your GP or for you know.um. Or the therapeutic environment rather than you know via video link and stuff arriving through. You know, pe, the people involved are all quite disconnected [Participant L, Male].

### Themes 4: ethics

This theme reflects participants’ views on the moral and ethical aspects of the treatment, including its alignment with personal values and transparency. Most found the treatment ethically acceptable:

Um I thought it was very ethical because everything was explained to me um well in advance, so I had plenty of time to do my own research. If you like, you know, find out what all the terminology meant…I’m in my own way, sort of with my own abilities, not just, you know, reading what you guys sent me because I wanted to. Not that I didn’t trust you. (short sharp laugh) I just needed to see what else I could find out. Um also…um there was, you know, there’s never any deception, nothing like that. So I thought it was ethical, I thought. um it’s something that I didn’t have any issues with participating in. And yeah, I’m. I’m glad I did it [Participant K, Female].

Um I have found that, um, the study was explained very, very clearly. [Researcher name] you’ve been absolutely brilliant, so patient and you’ve always sort of clearly explained how to how to set things up. And um…and I’ve always felt I could ask questions or if I’ve had any anxiety. So… And I’ve felt very well supported um throughout. So I have had absolutely no have had no qualms and I think it’s so important to do research around such a serious illness um…or disease or whatever. And and it’s really important and it’s life-saving research, really [Participant AC, Female].

Though one participant raised concerns about environmental issues:

Mmm. I’d say it’s very ethical apart from the the pads and the plastic that the pads come in, uhm the device itself is very ethical and you know it’s…Yeah, it’s… just the pads and the plastic [Participant I, Female].

### Themes 5: gratitude

This fifth main theme was gratitude with subthemes: (5a) appreciation for participation, (5b) feeling supported, (5c) interest in study outcomes, and (5d) hope.

### Subtheme 5a: appreciation for participation

Participants expressed gratitude for the opportunity to participate in the study, emphasizing how meaningful or fulfilling the experience was for them:

[…] I’m. I’m extremely grateful for, for, for being given the chance to try that out. As I say, nothing is ever written. Nothing useful is ever been offered to me, so this was the most useful thing I’ve I’ve ever had, really [Participant A, Female].

I just want to say to you thank you because I don’t know how you find me. I don’t know how uhm, but I’m so lucky to find you. I do this I did this treatment. I’m really happy because I was so upset in depression like and I was thinking, ok, it’s gonna take like one year more. But it didn’t happen. I was sore, feeling ashamed to my family, I was like…so much breaking, was looking uglier. Now I take care of me. I can go out. is it’s meaning a lot to me Maybe you can’t feel it but it’s meaning a lot. A lot to me. I’m really feeling happy [Participant F, Female].

### Subtheme 5b: feeling supported

Some participants expressed feeling emotionally supported and valued because they were included in the study and personally supervised by the study team:

(Sigh)You know, it felt like. Most of the time I was your only…You know… Whatever the word is, but like you’re very good at. Yeah, making made me feel like I’m your priority in that time. And I think that’s really important. And although I’m sure you’re juggling lots of people who are on this…Yeah, it’s, yeah. Just a lot of gratitude. Really just… [Participant T, Male].

[…] And it was fine. I’ve always felt treated with respect by yourself and and any… information about from the = University of East London = and was very impressed. I always felt as if I was… cared for and I was really appreciative of the fact that doing the sessions I had supervision, cause obviously being bipolar, I was a bit worried that it might send me my manic, but no very ethical, I am very happy and very appreciative for the staff who have helped me along the way of doing this [Participant Z, Female].

### Subtheme 5c: interest in study outcomes

Some participants showed their willingness to see the study outcomes:

Though I’d I’d love to see. As as you’ve you’ve promised to you know, sort of involve me in. um some of the information about the results and so on, so that I’m very interested in that [Participant L, Male].

I just can’t wait to see what the results are and I hope you’re successful with it. It’s, I hope finds you know, it’s, people can benefit from this being remote, so yeah [Participant AE, Female].

### Subtheme 5d: hope

This subtheme captures participants’ sense of hope and motivated attitude toward the treatment:

Um really helpful when when you’re in a situation where you feel like there isn’t any hope, Um…The each given me great hope. It’s. It’s not magic, but um it really given me a a step up on the ladder to climbing out to treat the depression, really [Participant Z, Female].

Initially I found it was helping me by giving me hope. I think it was like this is something else I’m trying which may work. I didn’t feel any benefit for the first few weeks and then once or twice after and towards the end of some sessions I started feeling some like a little uplift in mood or felt as though there was some mild positive effects [Participant X, Male].

### Theme 6: comparison to medications

This theme represents participants’ evaluations of the treatment in comparison to other methods, particularly medications, with two subthemes: (6a) side effects and tolerability, and (6b) effectiveness of tDCS vs. medications.

### Subtheme 6a: side effects and tolerability

Some participants compared tDCS with medication, noting how it is less invasive than medication and has fewer side effects:

I mean, obviously if you sort of take medication, you have this sort of quite it’s how it’s quite has quite quick impact, but also similarly with side effects and things. So it’s much more tolerable than that. And even though the change might feel a bit more subtle, it’s actually feels better to do something like that then know that [Participant B, Female].

### Subtheme 6b: effectiveness of tDCS vs. medications

Some other participants compared the effectiveness of tDCS to medication:

I think they were very helpful. because I tried different medications before and. They didn’t lift my mood like tDCS has done. So that’s the thing. I can relate my mood change to mostly. So I think it has been very helpful [Participant M, Female].

## Discussion

Patient acceptance of interventions positively influences the treatment process, resulting in improved outcomes. Measures such as treatment attrition rates have often been used to evaluate acceptability; however, these do not adequately capture its complex and multidimensional nature [5]. Qualitative methods are crucial for exploring complex situations and extending the scope of research, particularly in fields involving intricate human interactions [24]. In this study, we explored the acceptability of home-based tDCS as a qualitative construct, applying Sekhon et al.'s [5] framework to a group of individuals with bipolar depression. tDCS is emerging as a novel non-invasive treatment for bipolar depression. It has been administered in a clinic or research centre, requiring frequent visits to a clinical setting and creating a potential barrier to access. However, due to its portability and safety, tDCS can be administered at home. Home-based tDCS was generally well tolerated and deemed highly acceptable, with only mild and transient side effects and no serious adverse events or mood switching observed [18]. The thematic analysis of participants’ interviews identified six main themes, which were consistent with the quantitative results [18]. Four themes of helpfulness, side effects, burden and gratitude aligned with those identified in the unipolar depression study [21], with the additional themes of ethics and comparison to medications emerging in this study. Overall, the themes reflected the high acceptability of tDCS treatment in bipolar depression and highlighted the considerable interest in innovative non-pharmacological treatments within this population.

The helpfulness theme reflected the multifaceted perceptions of participants regarding the impact of home-based tDCS on their symptoms. This theme revealed a range of experiences and attitudes toward the treatment, including its perceived effectiveness, acceptability, and novelty. The majority of participants reported noticeable improvement in their depressive symptoms, highlighting the personal and perceived impact of the treatment on their well-being. Several participants reported unexpected and transformative benefits, describing outcomes that exceeded their initial scepticism or modest expectations. This suggests that the influence of expectations on tDCS outcomes may not always follow a linear pattern. A few participants described their experience of improvement as “gradual”, and a few described feeling that they experienced limited or no improvement. Participants frequently emphasized the innovative nature of tDCS and expressed appreciation for its departure from traditional pharmacological or psychotherapeutic approaches.

Acceptability is a dynamic construct shaped by attitudes, perceptions, and the novelty of interventions [25]. The perceived novelty of new health interventions further enhances their acceptance [26]. In this study, participants found the treatment highly acceptable, regardless of its effectiveness, reflecting the importance of acceptability in ensuring the successful implementation of innovative treatments [27]. However, some participants expressed uncertainty about whether the observed improvements were solely due to tDCS or influenced by other factors, such as lifestyle changes or concurrent treatments.

The second main theme, side effects, captures participants’ experiences with the physical sensations and overall tolerability of home-based tDCS. Reported side effects were mild, temporary, and did not deter participants from continuing treatment, emphasizing the intervention’s high tolerability. Commonly mentioned effects, such as itching, tingling, and slight redness of the skin, were anticipated and explained beforehand as typical side effects of tDCS [23]. These side effects diminished quickly post-session or as participants became accustomed to the treatment over time. In some cases, these physical sensations were viewed positively, as participants interpreted them as evidence of the treatment’s activity, which may reinforce perceptions of its efficacy [28]. Importantly, participants universally deemed the treatment tolerable and non-disruptive to their daily lives. The absence of severe or lasting side effects aligns with prior research on the safety profile of tDCS, which consistently highlights its minimal risk of adverse events [12, 18, 19, 29]. Transparent communication about potential side effects and preparing participants for minor discomforts before the start of treatment are essential for fostering trust, improving adherence, and enhancing the overall treatment experience by helping patients understand the risks and benefits of the intervention [30]. When concerns are minimized, patients are more likely to engage consistently with the treatment plan [31]. The findings further support the acceptability and perceived efficacy and safety of home-based tDCS, as reported by participants in this study.

The theme of burden explores the challenges participants faced during home-based tDCS treatment, focusing on usability, time commitment, and treatment setting. Despite some minor obstacles, the treatment was generally considered manageable and convenient. In terms of technical usability, most participants found the technology user-friendly and straightforward. These impressions are specific to the device used in this study and may not generalize to other tDCS systems with different interfaces. However, a few experienced minor issues, such as app setup, which occasionally disrupted their readiness to begin sessions. The time commitment of the treatment was widely regarded as reasonable and accommodating, with many participants appreciating the short session durations and their ability to seamlessly integrate them into daily routines. While some participants initially reported difficulties fitting the sessions into their busy schedules, similar to findings in unipolar depression [21], they were ultimately able to accommodate to the sessions successfully. The manageable nature of the sessions reduced the perceived effort required to engage with the treatment [5]. For some, the structured routine provided by the sessions was perceived as a positive aspect, fostering a sense of regularity and purpose, which reflects enhanced self-efficacy. Participants expressed empowerment in their ability to incorporate tDCS sessions into their daily lives, demonstrating confidence in balancing both treatment and personal responsibilities [32].

The majority of participants emphasized the convenience of receiving tDCS at home, which is consistent with the findings in unipolar depression study [21]. A significant advantage of home-based tDCS protocols is the autonomy to schedule sessions according to personal preferences, reducing the need for travel and allowing participants to remain in a comfortable, familiar environment. This flexibility enabled them to maintain their routine activities and responsibilities while receiving treatment. These findings highlight the importance of intuitive design and user-friendly interfaces in facilitating the seamless integration of tDCS into participants’ daily routines. However, two participants expressed a preference for in-person sessions, citing the potential benefits of face-to-face interaction with healthcare professionals and the structured environment of a clinical setting.

The ethics theme reflected participants’ perspectives on the ethical aspects of home-based tDCS. All participants found the treatment to be transparent and aligned with their personal values, highlighting key ethical principles such as clear communication, informed consent, and respect for autonomy, which reflect the emphasis on user empowerment in tDCS ethics [33]. Additionally, the opportunity to conduct independent research further reinforced participants’ confidence in the process. There were only two ethical concerns regarding the tDCS device, one participant raised concerns about environmental issues, and one regarding the device being delivered from abroad. Ethics did not emerge as a main theme in the unipolar depression study, possibly because participants’ assumptions were fulfilled or the information aligned with their values [21]. In this study, although the information was also aligned with participants’ values, many participants engaged with the ethical questions in different ways. As a result, ethics emerged as a distinct theme. The additional two main themes, gratitude and comparison with medications, have not been described in Sekhon et al.'s [5] framework but were identified in this study due to their frequent mention by participants regarding the treatment.

The fifth main theme was gratitude, highlighting participants’ positive emotional responses to their involvement in the study and treatment, emphasizing appreciation, emotional support, and hope. Many participants expressed deep gratitude for the opportunity to participate, viewing the study as meaningful and impactful. This sentiment reflects the value they placed on being given access to an innovative treatment, especially in contexts where traditional options had been unhelpful. Gratitude fosters positive emotions and enhances individuals’ sense of connection and resilience, particularly when engaging with meaningful and supportive experiences [34–36]. Participants also felt supported and valued through their interactions with the same research team member, which contributed to a sense of personal care and emotional encouragement. This level of support likely increased their engagement and overall satisfaction with the treatment process. The experience of being observed and cared for can greatly enhance patients’ emotional security and overall well-being. Such personalized interactions help patients feel valued, supported in their treatment journey, and build greater trust in their care process [37, 38]. Additionally, participants expressed a strong interest in the study outcomes, showing enthusiasm about the potential benefits of the treatment for others and recognizing the impact of their contributions. Hope emerged as a key subtheme, with participants explaining how engaging in the treatment boosted their sense of optimism, motivated them to explore new treatment options, and actively work toward overcoming depression. High level of hope in patients has been shown to enhance treatment adherence. Those who had a stronger sense of hope were less likely to discontinue their treatment and were more likely to view their treatment as effective and worthwhile [39, 40]. In the unipolar depression study, ‘support, feeling held and contained’ was identified as the main theme, emphasizing the benefits of researcher presence and the balance between safety and anxiety [21].

Although participants had not been asked to compare tDCS with other medications, many spontaneously made comparisons to pharmacological treatments, focusing on side effects, tolerability, and effectiveness. Participants frequently described tDCS as less invasive, more tolerable, and in some cases, associated with better improvement. This theme had not emerged in the unipolar depression study [21].These reflections represent subjective perceptions rather than objective clinical comparisons. While such perspectives align with previous findings on the tolerability of tDCS [12, 29], they should be interpreted as individual impressions rather than evidence of superiority over standard treatments.

Limitations include a lack of a sham treatment arm, as all participants received active tDCS in an open-label design, leaving no opportunity to compare participants’ experiences of tDCS with a sham condition. Another limitation is that the study did not control for the types of medications participants were using. While participants were required to maintain a stable dosage of mood-stabilizing medication for at least two weeks or abstain from medication for the same duration, mood stabilizers such as lithium and lamotrigine exert their effects through the modulation of cortical excitability, which may influence tDCS efficacy [15]. Some participants expressed uncertainty about whether observed improvements were due solely to tDCS or influenced by other factors. This highlights the challenge of isolating the specific effects of tDCS and raises concerns about confounding variables, such as types of medications or concurrent psychotherapy, that were not controlled for. The study included a relatively small sample size with predominantly white ethnicity and a greater proportion of female participants, limiting the generalizability of the findings. Personalized interactions with the same research team member were noted as a strength, but they may have also contributed to the high response and remission rates [18]. Most participants had been recruited through online advertisements, which may limit the generalizability of the findings to individuals less familiar with digital platforms. Participants were allowed to engage in quiet activities of their choice during tDCS sessions, such as reading, using a device, or sitting quietly. While this approach reflects real-world use, it may have introduced inter-subject variability due to task-dependent plasticity effects [41] and state-dependent effects of tDCS [42]. In our previous open-label study [17] and randomized controlled trial [19] of home-based tDCS in unipolar depression, participants were asked to sit or lie down without engaging in distracting tasks during stimulation.

## Conclusion

We conducted a qualitative analysis of the acceptability of home-based tDCS protocols with real-time supervision for bipolar depression, based on Sekhon et al.'s [5] framework of acceptability. Six main themes emerged: helpfulness, side effects, burden, and ethics, aligning with the proposed components of acceptability. Additional themes of gratitude and comparison to medications were identified in the present sample in bipolar depression. Overall, the themes reflect participants’ general high acceptability of tDCS treatment in bipolar depression. Qualitative research offers a deeper understanding of human behavior and the contextual factors that shape outcomes. This approach is especially valuable in healthcare, where it helps evaluate patient experiences, identify barriers to treatment, and improve service delivery [43]. Future studies should incorporate a sham treatment control group to provide deeper insights into how beliefs and attitudes toward treatment impact clinical outcomes.

## Electronic supplementary material

Below is the link to the electronic supplementary material.

Supplementary Material 1

## Data Availability

The anonymised datasets used and/or analysed during the current study are available from the corresponding author on reasonable request.
