# Supplementary material for: Acceptability of home-based transcranial direct current stimulation (tDCS) in bipolar depression: thematic analysis of individual views
Source: BMC Psychiatry. 2025 May 26;25:549. doi: 10.1186/s12888-025-06948-4 (PMC12107877; doi:10.1186/s12888-025-06948-4)
Supplement: Supplementary file 1 — Supplementary Material 1 [file 12888_2025_6948_MOESM1_ESM.docx]

**Supplementary Materials**

**Title:**

Acceptability of Home-Based Transcranial Direct Current Stimulation (tDCS) in Bipolar Depression: Thematic Analysis of Individual Views

**Authors:**

Hakimeh Rezaei ^a, b, c^, Rachel D. Woodham ^c^, Ali-Reza Ghazi-Noori ^c^, Philipp Ritter ^a, b^, Michael Bauer ^a^, Allan H. Young ^b,^ ^e, f^, Elvira Bramon ^d^, Cynthia H.Y. Fu ^b, c, e^

**Affiliations:**

1. Department of Psychiatry and Psychotherapy, Faculty of Medicine, Technische Universität Dresden, Dresden, Germany
2. Institute of Psychiatry, Psychology and Neuroscience, King’s College London, London, UK
3. Department of Psychology, University of East London, London, UK
4. Department of Psychiatry, University College London, London, UK
5. National Institute for Health Research Biomedical Research Centre at South London and Maudsley NHS Foundation Trust, King's College London, London, UK
6. South London and Maudsley NHS Foundation Trust, Bethlem Royal Hospital, Beckenham, UK

Table of Contents

[Figure 1. tDCS headset and electrode positioning diagram 3](#_Toc195283773)

[Inclusion and exclusion criteria 4](#_Toc195283774)

[Quantitative thematic analysis-steps and process 5](#_Toc195283775)

[Table 1. Demographic and clinical data 7](#_Toc195283776)

[Table 2. Coding summary 8](#_Toc195283777)

[Supplementary reference 10](#_Toc195283778)

**Figure 1. tDCS headset and electrode positioning diagram**.

(A) Figure depicting tDCS headset (Flow Neuroscience, Sweden). (B) The targeted stimulation locations at F3 and F4 are shown, with the anode in blue and the cathode in red, according to the International 10-20 EEG System.

# **Inclusion and exclusion criteria**

Inclusion criteria included:

1. capable of giving informed consent
2. male and female
3. adults aged 18 or older
4. diagnosis of bipolar disorder, defined by Diagnostic Statistic Manual of Mental Disorders, Fifth Edition (DSM-5)[1], in a structured clinical assessment using the Mini-International Neuropsychiatric Interview (MINI; Version 7.0.2) [2]
5. having at least a moderate severity of depressive symptoms as measured by a minimum score of 18 on Montgomery-Åsberg Depression Rating Scale (MADRS) [3]
6. taking a stable dosage of mood-stabilizing medication for a minimum of two weeks or not taking any medication for a minimum of two weeks.

Exclusion criteria included:

1. any concurrent psychiatric disorder, including obsessive compulsive disorder
2. significant suicide risk, assessed by Suicidality module of the Mini-International Neuropsychiatric Interview [2], Montgomery-Åsberg Depression Rating Scale and 17-item Hamilton Depression Rating Scale (HDRS-17) [4]
3. symptoms of mania or hypomania as measured by score greater than 8 on Young Mania Rating Scale (YMRS) [5]
4. exclusion criteria for tDCS, including having a scalp or skin conditions, metallic implants
5. history of epilepsy
6. a history of seizures with loss of consciousness
7. a history of neurological disorder or history of migraines.

# **Quantitative thematic analysis-steps and process**

Thematic analysis typically involves the following six steps:

1. **Familiarization with the Data**: Reading, transcribing, and making notes in the transcripts to become immersed in the data and identify preliminary patterns.
2. Generating Initial Codes: Systematically coding relevant features in the data that address the research question.
3. Searching for Themes: Organizing related codes into broader themes that capture significant patterns across the dataset.
4. Reviewing Themes: Validating and refining themes by checking their coherence and alignment with the dataset.
5. Defining and Naming Themes: Clearly defining the scope of each theme and providing concise, descriptive labels.
6. **Producing the Report:** Synthesizing findings and presenting themes with supporting examples, such as participant quotes, to illustrate their relevance [6].

After outlining the thematic analysis framework, the steps undertaken in this study are described below:

- Stage 1: The first stage involved verbatim transcription of the interviews, following a standardized qualitative data preparation and transcription protocol [7]. This protocol ensured consistent formatting, verbatim transcription, and the inclusion of non-verbal cues for accurate data representation. The transcripts were read multiple times to deepen familiarity with the content, and all data were thoroughly reviewed. During this process, key points, initial thoughts, and potential patterns were noted as preliminary observations.
- Stage 2: In the second stage, the data was systematically coded by breaking it into manageable segments. Initial codes were generated based on both their relevance to the research question and emerging patterns within the data. Each segment of the text was examined, and a descriptive label (code) was assigned.
- Stage 3: The third stage involved organizing the initial codes into broader themes that reflected recurring patterns or key concepts within the data. Related codes were grouped together, and potential themes were identified to represent underlying meanings in the dataset. A thematic map was created to visually depict the connections between themes and sub-themes, illustrating how they related to each other and to the research question.
- Stage 4: In this stage, the identified themes were reviewed to ensure that they accurately reflected the data and were coherent, distinct, and relevant. Any redundant or overlapping themes were refined or merged, and sub-themes were created where appropriate to provide additional structure. These themes were discussed with the supervisor and other research team members to ensure consistency and to test their coherence.
- Stage 5: Each theme was carefully defined and named to reflect its relevance to the research question. Where necessary, the subthemes were identified to highlight specific aspects within broader themes.
- Stage 6: The final stage involved writing the report, which presented the findings with clear explanations of each theme and its relevance to the research question. Representative quotes from participants were selected to illustrate and support the identified themes.

# **Table 1. Demographic and clinical data**

| Participant Characteristics |  |
| --- | --- |
| Total number (Female) | 35 (26) |
| Mean Age (years) | 47.37 ± 13.78 |
| Age range (years) | 24-76 |
| Years of education | 16.63 ± 2.41 |
| IQ | 101.57 ± 9.85 |
| Ethnicity | |
| White British | 27 |
| White Romanian-British | 1 |
| White Norwegian | 1 |
| White Ukrainian | 1 |
| Other White | 1 |
| Asian Turkish | 1 |
| Asian British | 2 |
| Caucasian | 1 |
| Clinical rating | |
| MADRS at baseline | 24.77 ± 2.72 |
| Duration of illness (years) | 16.71 ± 11.20 |
| Duration current depressive episode (weeks) (range) | 54.60 ± 111.83 |
| Previous number of episodes | 17.97 ± 20.69 |
| Treatments during trial | |
| Taking mood stabilizer and other medications (%) | 29 (83) |
| Taking antidepressant medication only (%) | 1 (2) |
| Taking no medication (%) | 4 (11) |
| Engaged in psychotherapy (%) | 7 (20) |

Categorial variables are presented as number of participants with percentage in parentheses

for treatment during trial. Mean values are presented with ± standard deviation. MADRS, Montgomery-Åsberg Depression Rating Scale. IQ was measured using Ammons’s Quick Test [8]

# **Table 2. Coding summary**

| **Themes** | **Subthemes** | **Category** | **Code summary** | **Example quote** |
| --- | --- | --- | --- | --- |
| Helpfulness | 1. effectiveness and acceptability 2. unexpected improvement 3. gradual improvement 4. novelty and un/certainty | acceptability  helpfulness  effectiveness | clear improvement vs. uncertainty about effectiveness; significant improvement vs. ineffectiveness or slight improvement;  acceptable;  unexpected effectiveness;  gradual effectiveness; | “Yeah, I found them very beneficial. My depression has definitely gone down. Uhm…I don't have this black cloud hanging over me. Uhm Yeah, they were very tolerable.”  “I I was really shocked and my sister  was, like, shocked. And she she told me, yeah, you just like look better and you record. I didn't even understand before my sister understood…“ |
| Side Effects | 1. side effects of the device and tolerability 2. temporary side effect or adapotion | side effect | tolerable treatment experience; no side effects vs. minimal discomfort;  minor or temporary effects | “No, not at all. I thought I might get headaches, but I didn't. No, absolutely fine.”  “Nothing I wouldn’t say any real side effects at all.” |
| Burden | 1. technical usability 2. time commitment 3. treatment setting | burden | effort technical challenge vs. easy and straightforward to use;  remote setting/ home setting vs. preference for human interaction; well organized; able to combine the sessions with duties; role of routine in enhancing recovery;  short and manageable vs. time consuming and difficult to fit in; | “Yeah, the duration was short enough that you can fit it in. Even in a busy…Um lifestyle. Like a half an hour session goes really quickly. Um you don't have to do very much, you just sit there.”  “No, we aren't only problem I had was with my first flow set uhm and that was remedied.”  “It's, I hope finds you know, it’s…people can benefit from this being remote, so yeah.” |
| Ethics | (No subtheme) | ethics | ethicality; approved; free choice; well explained; clarity of study information; predictable; no interference; informative; ethical concern; morally positive; concern about device delivered from abroad or plastic waste. | “Extremely ethical…Uhm you know that. I knew that I could withdraw from the trial at anytime.”  “Umm yeah, I kept complete trust and faith in.  The trial and the way it was run. Yeah, very moral. Very ethical.” |
| Gratitude | 1. appreciation for participation 2. feeling supported 3. interest in study outcomes 4. hope | (no category) | positive feedback; convenient experience; willingness to recommend; worth trying; life changing; positive feedback on app; interest to know the result; gratitude and appreciation; feeling support. | “I always felt supported and if I if I needed any help, I knew who to turn to…”  “Um just…I really appreciate all the time you've taken (short laugh) to sort of take me through this process. …happy to be part of something that has made such a difference.”  “I just can't wait to see what the results are and I hope you're successful with it.” |
| Comparison to Medications | 1. side effects and tolerability 2. effectiveness of tDCS vs. medications | (no category) | tDCS perceived more tolerable/gentler than medications; better than medications; less invasive than medications. | “I suppose I feel like my mood is definitely lifted. I felt better than I felt in a long long time. Without maybe the side effects of medication.”  “[…] for decades and decades and I'm doing something new that's doesn't feel as invasive as medication to me feels like a a kind of a a moral positive…” |

# **Supplementary reference**

1. American Psychiatric Association: Diagnostic and Statistical Manual of Mental Disorders. American Psychiatric Association. 2013.

2. Sheehan D V, Lecrubier Y, Sheehan KH, et al: The Mini-International Neuropsychiatric Interview (M.I.N.I.): the development and validation of a structured diagnostic psychiatric interview for DSM-IV and ICD-10. J Clin Psychiatry 59 Suppl. 1998; 20:22-33;quiz 34-57

3. Montgomery SA, Åsberg M. A New Depression Scale Designed to be Sensitive to Change. Br J Psychiatry.1979;134:382–389. https://doi.org/10.1192/bjp.134.4.382

4. Hamilton M. A Rating Scale for Depression. J Neurol Neurosurg Psychiatry.1960; 23:56–62. https://doi.org/10.1136/jnnp.23.1.565.

5. Young RC, Biggs JT, Ziegler VE, Meyer DA. A Rating Scale for Mania: Reliability, Validity and Sensitivity. Br J Psychiatry.1978;133:429–435. <https://doi.org/10.1192/bjp.133.5.429>

6. Braun V, Clarke V. Using thematic analysis in psychology. Qual Res Psychol. 2006;3:77–101. https://doi.org/10.1191/1478088706qp063oa

7. McLellan E, MacQueen KM, Neidig JL. Beyond the Qualitative Interview: Data Preparation and Transcription. Field methods.2003;15:63–84. https://doi.org/10.1177/1525822X02239573

8. Ammons RB, Ammons CH. The Quick Test (QT): Provisional Manual,. Psychol Rep. 1962;11:111–61. https://doi.org/10.1177/003329416201100106
